# Modes of Neuronal Calcium Entry and Homeostasis following Cerebral Ischemia

**DOI:** 10.4061/2010/316862

**Published:** 2010-11-01

**Authors:** J. L. Cross, B. P. Meloni, A. J. Bakker, S. Lee, N. W. Knuckey

**Affiliations:** ^1^Centre for Neuromuscular and Neurological Disorders, Australian Neuromuscular Research Institute, University of Western Australia, WA 6009, Australia; ^2^Department of Neurosurgery, Sir Charles Gairdner Hospital, WA 6009, Australia; ^3^School of Biomedical, Biomolecular and Chemical Sciences, University of Western Australia, WA 6009, Australia

## Abstract

One of the major instigators leading to neuronal cell death and brain damage following cerebral ischemia is calcium dysregulation. The neuron's inability to maintain calcium homeostasis is believed to be a result of increased calcium influx and impaired calcium extrusion across the plasma membrane. The need to better understand the cellular and biochemical mechanisms of calcium dysregulation contributing to neuronal loss following stroke/cerebral ischemia is essential for the development of new treatments in order to reduce ischemic brain injury. The aim of this paper is to provide a concise overview of the various calcium influx pathways in response to ischemia and how neuronal cells attempts to overcome this calcium overload.

## 1. Introduction

### 1.1. Cerebral Ischemia and Neuronal Cell Death

Cerebral ischemia results in a reduced blood supply to brain tissue, causing oxygen-glucose deprivation and ATP production failure. The resulting energy crisis can trigger a cascade of detrimental biochemical and physiological events leading to acute or delayed cell death [1]. The lack of ATP synthesis causes the loss of ion homeostasis, resulting in membrane depolarisation and release of the neurotransmitter glutamate. High extracellular glutamate causes excitotoxicity resulting in NMDA, AMPA, and kainic acid receptor activation allowing the influx of calcium, sodium, and zinc ions into the cell. If ATP synthesis inhibition is sustained, acute cell death occurs. If ATP synthesis is transiently or mildly inhibited, delayed cell death can occur. In acute cell death, excessive intracellular calcium activates harmful phospholipases, endonucleases, and calpains causing cell organelle and membrane breakdown, leading predominantly to a necrotic-like cell death. In delayed cell death, the initial or milder periods of excitotoxicity can trigger a range of cellular disturbances such as oxidative stress, protein synthesis/folding disturbances, mitochondrial dysfunction, and altered cell signalling. The accumulation of these cellular disturbances can eventually cause a secondary rise in intracellular calcium and the activation of cell death pathways (apoptosis, necrosis, autophagy, and necroptosis), ultimately leading to the demise of the neuron.

### 1.2. Cerebral Ischemia and Calcium Dysregulation

Although the exact mechanisms underlying how neuronal calcium disturbances lead to cell death have not been fully elucidated, the major pathways responsible for calcium overload during and following ischemia are better characterised. Ultimately, calcium dysregulation and overload occur when there is a disequilibrium concerning homeostatic pathways controlling calcium influx, efflux, and release from intracellular organelles. 

The major pathways involved in ischemia-associated neuronal calcium influx are the glutamate receptor channels (divided into two subtypes: the ionotropic receptors NMDA, AMPA, and KA and the metabotropic receptors mGluR), voltage-dependent calcium channels (VDCCs), and the sodium calcium exchanger (NCX) [2, 3]. More recently, transient receptor ion channels (TRPM and specifically TRPM7) [4], acid-sensing ion channels (ASIC) [5], and inward excitotoxic injury current (*I*_EIC_)–calcium-permeable channels [6] have also been implicated in calcium influx. In addition, the release of calcium from organelles, namely the mitochondria and endoplasmic reticulum (ER), can also contribute to neuronal intracellular calcium overload following ischemia [7, 8].

**Figure 1:**
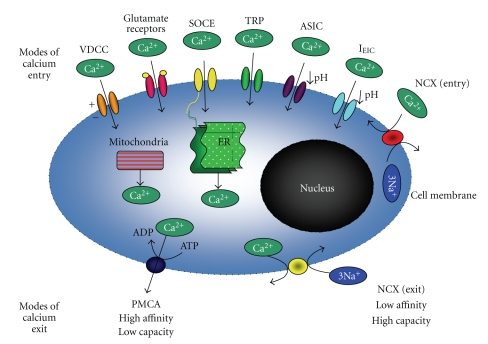
Modes of calcium entry and exit into neurons following cerebral ischemia. Modes of calcium entry (top of cell diagram) are VDCCs (voltage-dependent calcium channel), glutamate receptors (NMDA, AMPA, KA, and mGluR), SOCE (store-operated intracellular calcium entry), TRP (transient receptor potential channels), ASIC (acid-sensing ion channels), I_EIC_ (inward excitotoxic injury current calcium-permeable channels), and NCX (sodium-calcium exchanger operating in entry mode). Calcium can also be sequestered intracellularly (middle of cell diagram) by the mitochondria and ER (endoplasmic reticulum). Modes of calcium exit (bottom of cell diagram) are PCMA (Calcium ATPase pump) and NCX (sodium-calcium exchanger operating in exit mode).

With respect to calcium efflux, there are only two known mechanisms: via the calcium ATP-ase pump (plasma membrane calcium ATPase pump (PMCA)) and through sodium calcium exchanger (NCX) exit mode activity [9] (Figure 1). Taken together, here lie a number of possible therapeutic targets to manipulate intracellular calcium levels after an ischemic episode and thereby reduce neuronal death. With this in mind, this paper will focus on providing a concise update of the major modes of calcium influx, efflux, and release from organelles following cerebral ischemia.

## 2. Modes of Neuronal Intracellular Calcium Entry following Cerebral Ischemia

### 2.1. Glutamate Receptors

Glutamate receptors are located on the cytoplasmic membrane of neurons and are activated following the binding of the neurotransmitter glutamate. Their main function following glutamate binding is to cause postsynaptic excitatory transmission; however, following ischemia their over stimulation can be damaging to neurons. Glutamate receptors can be divided into two broad groups based on selective affinity for different agonists: (1) Ionotropic glutamate receptors which include N-methyl-D-aspartic acid (NMDA) receptors, *α*-amino-3-hydroxy-5-methyl-4-isoxazolepropionic acid (AMPA) receptors and kainic acid (KA) receptors; and (2) the metabotropic glutamate receptors (mGluR), which are activated selectively by quisqualate (an agonist of mGluR). Metabotropic receptors, unlike ionotropic receptors, do not form an ion channel but rather interact with other receptors on the cell membrane and are linked to G-protein activation of phospholipase C, which converts phosphotidylinositol bisphosphate in the cell membrane to inositol trisphosphate and diacylglycerol. Inositol trisphosphate acts to release calcium from the ER, whilst diacylglycerol activates protein kinase C (PKC) which mediates many effects. The release of calcium ions is required for activation of calcium-dependent PKC isoforms; likewise, PKC can phosphorylate proteins that can then alter calcium signalling.

With respect to ionotropic glutamate receptors during ischemia, excessive activation of NMDA and AMPA glutamate receptors is a major source of calcium influx [10–14]. Likewise, activation of certain metabotropic glutamate receptor subtypes can cause the release of calcium from the ER [15].

#### 2.1.1. NMDA Receptors

The NMDA receptor is a nonspecific cation channel with a high affinity for calcium ions. It is comprised of a four peptide subunit structure with several different subunits being identified (NR1, NR2A-D, and NR3) [16]. Activation of this receptor by extracellular glutamate results in neuronal membrane depolarisation and VDCCs-mediated calcium influx, as well as calcium influx through the channel itself. Hence, excessive activation of these receptors and the resulting calcium-associated influx would have direct and indirect damaging effects within the cell. Moreover, it appears that the subcellular location of NMDA receptors, which can be either synaptic (segregated on and around the synapse) or extrasynaptic (located outside of the synaptic cleft) plays an important role in terms of neuronal fate following activation. NMDA receptors which contain the NR2A subunits have been shown to be located primarily in the synapse, whilst those receptors containing the NR2B subunits are located predominantly in the extra-synaptic zones of neurons [17].

**(1) Synaptic NMDA receptor activation**

Recent evidence has shown that activation of synaptic NMDA receptors is associated with a prosurvival response in neurons. This response, which has mainly been characterised using cultured neurons, is trigged by a mild nondamaging level of NMDA receptor activation. The prosurvival response is associated with the upregulation of prosurvival proteins (e.g., BCL6, BTG2) and downregulation of prodeath proteins (e.g., CASP8AP2, DIDO1) [18].

**(2) Extrasynaptic NMDA receptor activation**

In contrast to synaptic NMDA activation, overstimulation of extra-synaptic receptors triggers a neuronal damaging signalling response. For example, stimulation of extra-synaptic NMDA receptors can mediate upregulation of the CLCA1 (calcium-activated chloride channel)and activation of p38 (mitogen-activated protein kinase p38) both of which contribute to neuronal death [18–20].

In addition to NMDA receptor subcellular location, receptor subunit composition is also important in determining neuronal fate following cerebral ischemia [21]. It has been demonstrated that following ischemia and NDMA receptor activation, NR2A and NR3 subunit containing receptors promote neuronal survival signalling pathways [14, 22, 23], while NR2B-containing receptors mediate neuronal death signals [24–28]. With respect to neuronal death signalling, this can involve interaction of the intracellular domain of the NR2B subunits with proteins such as DAPK1 (death-associated protein kinase 1) and PSD-95 (postsynaptic density protein 95) or downstream activation of proteins, such as SREBP-1 (sterol regulatory element-binding protein-1) [27, 28]. DAPK1 and SREBP-1 activation is associated with apoptotic cell death, while PSD-95 signalling is associated with nitric oxide production. The prosurvival effects mediated by NR2A and NR3 subtypes are less well characterised, but are probably associated with the regulation of neuroprotective and neurodamaging proteins as described in Zhang et al. [18].

#### 2.1.2. AMPA Receptors

AMPA receptors are nonspecific cation channels that consist of four subunits (GluR1-4), with receptor permeability to calcium dependent on the configuration of the subunits [29, 30]. AMPA assemblies that contain the GluR1, GluR3, and GluR4 subunits are permeable to calcium ions, while GluR2 subunit containing assemblies are impermeable to calcium [11, 30]. To this end, downregulation of GluR2 protein expression following cerebral ischemia is associated with intracellular calcium and/or zinc influx and neuronal degeneration [13]. In addition, it is likely that activation of low calcium-permeable AMPA receptors exerts indirect neurodamaging effects following ischemia due to their role in VDCCs-mediated calcium influx.

#### 2.1.3. KA Receptors

KA receptors consist of a four subunit structure containing a combination of one or more of five different subunits (KA1, KA2, and GluR5-7). Receptor subunit composition determines receptor permeability and function. KA receptors are normally permeable to sodium and potassium ions and generally not permeable to calcium [31]. Their role in neuronal fate following ischemia is not well understood, but there is evidence that their activation can stimulate survival pathways through regulation of the inhibitory neurotransmitter, *γ*-aminobutyric acid (GABA). For example, it is considered that the post-synaptic KA receptor-mediated release of GABA activates GABA receptors, causing inhibition of ischemia-induced NMDA overactivation [32–36].

#### 2.1.4. Metabotropic Glutamate Receptors

Metabotropic glutamate receptors can be divided into three different families with subtypes for each group consisting of: Group I (mGluR1, mGluR5), Group II (mGluR2, mGluR3), and Group III (mGluR4, mGluR6-8). Due to metabotropic receptor-mediated release of calcium from the ER these receptors can contribute to increased intracellular calcium following ischemia. However, it has also been demonstrated that metabotropic glutamate receptor agonists can be protective following ischemia [37–41].

### 2.2. Voltage-Dependent Calcium Channels

Voltage-dependent calcium channels (VDCCs) are a type of transmembrane ion channel found in excitable cells and are composed of four homologous *α*1 transmembrane subunits which form a calcium-permeable pore along with *α*_2_ *δ*, *β*_1-4_, and *γ* auxiliary subunits which function in modulating the channel complex [42]. There exist several structurally related subtypes, including L-type, N-type, P/Q-type, and T-type. During an ischemic event, neuronal membrane depolarisation results in the activation of these channels and intracellular calcium influx. 

#### 2.2.1. L-Type VDCCs

L-type VDCCs (otherwise known as long-lasting or DHP receptors) are commonly found on dendritic neurons and, when activated, trigger calcium influx and the expression of genes leading to cell survival [10]. However, during the early phases of ischemia and reperfusion, L-type channel activation is likely to contribute to calcium dysregulation and cell death, as their inhibition before or early after cerebral ischemia is neuroprotective [43, 44]. Interestingly, in the later stages after ischemia/reperfusion L-type channels are downregulated [43], a process that is thought to contribute to delayed neuronal death, as the administration of channel agonists in late postischemia settings is neuroprotective [43, 45].

#### 2.2.2. N-Type VDCCs

N-type VDCCs (otherwise known as neural) play a primary role in neurotransmitter release from the presynaptic terminal via the influx of calcium after depolarisation. The toxin *ώ*-conotoxin is a specific blocker of these channels and is regularly used to study their function and mechanisms. Early studies [46–49] revealed that a synthetic peptide, SNX-111 (a selective N-type VDCCs blocker), was found to be highly neuroprotective following global cerebral ischemia, suggesting that N-type calcium-channels play an important role in calcium associated ischemia and neuronal injury.

#### 2.2.3. P/Q-Type VDCCs

P/Q-type VDCCs (or Purkinje) are found mainly in the cerebellum and are involved in presynaptic neurotransmitter release. Blockade of these channels with the *ώ*-agatoxin has been shown to reduce brain infarcts following focal cerebral ischemia [50].

#### 2.2.4. T-Type VDCCs

T-type VDCCs (or transient) are associated with low-voltage activity in the brain and activate when the neurons are at rest (~−60 mV) allowing small amounts of calcium influx, which have been shown to benefit signal amplification. Specific inhibitors of these channels have been shown to dramatically reduce neuronal damage in hippocampal slice cultures following oxygen-glucose deprivation [51].

### 2.3. Transient Receptor Potential Channels

Transient receptor potential (TRP) channels are a family of cation channels which are nonselective for ions such as magnesium, sodium, and calcium. TRP channels consist of six transmembrane segments with pore formation between segments 5 and 6 [52]. In mammals, they can be divided into six subfamilies: TRPC (canonical), TRPV (vanilloid), TRPM (melastatin), TRPP (polycystin), TRPML (mucolipin), and TRPA (ankyrin) [53]. TRP channels have been associated with many different diseases and have been implicated in some kidney and heart disease whilst also playing an important role in cerebral ischemia [4, 54].

Of particular interest for this paper are the TRPM and TRPC family, members. Within the TRPM family there exists eight subtypes (TRPM1-8). Of these subtypes the TRPM7 and to a lesser extent TRPM2 members are the most important with respect to ischemia-induced neuronal calcium influx [55], as these receptors are activated by oxidative mechanisms which are increased during ischemia and result in large intracellular calcium influxes. Downregulation of TRPM7 is neuroprotective following global ischemia [56].

TRPC channels can be divided into four main subgroups (TRPC1, TRPC2, TRPC3/6/7, and TRPC4/5), and they are thought to play an important role in regulating the refilling of the intracellular calcium stores after phospholipase C-induced calcium release. After calcium release, a proportion of the released calcium is pumped out of the neuron due to the activity of the plasmalemmal calcium extrusion systems, making it unavailable for reuptake by the calcium store. The TRPC channels are activated by calcium store depletion, and the ensuing calcium entry provides the calcium necessary for complete refilling of the calcium store [57, 58]. It has been shown that TRPC channels, in particular the TRPC1 form, have increased expression in hippocampal organotypic slices following glutamate exposure, and their activation contributes to neuronal cell death [59]. It has also been shown that TRPC1 is activated by the metabotropic glutamate receptor mGluR1 [60]. Hence, it is reasonable to conclude that these receptors are likely to contribute to neuronal intracellular calcium influx following ischemia.

### 2.4. Acid-Sensing Ion Channels

Acid-sensing Ion Channels (ASIC) are nonselective ion channels, which are activated in response to decreased extracellular pH. These trimeric channels consist of one or more of six different subunits (ASIC1a, ASIC1b, ASIC2a, ASIC2b, ASIC3, and ASIC4), which vary in their response to pH levels. In the event of cerebral ischemia, the resulting extracellular pH decrease triggers ASIC channel opening, allowing calcium to enter neurons [61]. It has been reported that pharmacological blockade or gene knockdown of ASIC in stroke models attenuates neuronal injury [62, 63]. Although both ASIC1a and ASIC2a subunits are found abundantly in the brain, ASIC1a containing channels are considered to play a major role in calcium-mediated ischemic brain injury [64]. Furthermore, it has been demonstrated that NR2B-containing NMDA receptors can activate the calcium/calmodulin-dependent protein kinase II (CaMKII) pathway causing the phosphorylation of the ASIC1a channel leading to acidotoxicity-induced cell death [65].

### 2.5. Sodium-Calcium Exchanger (Calcium Entry Mode)

The sodium-calcium exchanger (NCX) is a bidirectional ion transporter, with a low affinity but high transporting capacity for calcium. Its structure consists of nine transmembrane segments which are involved in binding and transportation of sodium and calcium ions and a large intracellular hydrophilic loop which functions to regulate NCX activity [66]. There exist three isoforms (NCX1, NCX2, and NCX3), all of which are expressed in the brain. Under normal physiological conditions, NCX's main function is to expel calcium out of the cell (forward or calcium exit mode) whilst concurrently transporting sodium into the cell by using the electrochemical gradient of the sodium ions. However, under certain pathological conditions such as cerebral ischemia, NCX can operate in reverse or calcium entry mode, promoting potentially damaging calcium influx [2, 67]. Here lies conflicting opinions as to whether NCX is neuroprotective or neurodamaging. 

One working model is that under severe ischemic conditions, the NCX operates in calcium entry mode and facilitates calcium-induced acute neuronal cell death. Under these conditions-blocking NCX activity is neuroprotective [68–70]. In contrast, during or after milder episodes of cerebral ischemia, which normally results in neuronal recovery of delayed neuronal death, the NCX operates in calcium exit mode in an attempt to restore calcium homeostasis [71–73]. To this end, it has been demonstrated that the proteolytic inactivation of NCX3 can occur following cerebral ischemia, rendering the channel inactive and resulting in reduced calcium efflux, contributing to calcium dysregulation and cell death [74].

### 2.6. Inward Excitotoxicity Injury Current (I_EIC_)—Calcium-Permeable Channel

A recent study [6] has described a novel calcium-permeable channel in cultured hippocampal neurons identified as the inward excitotoxic injury channel (I_EIC_), which the authors believe is also responsible for glutamate-induced extended neuronal depolarisation (END) and calcium-mediated excitotoxicity. Based on *in vitro* experimental studies, the I_EIC_ is activated after an excitotoxic insult, and once activated results in sustained neuronal calcium entry. Further investigations showed that blocking of the I_EIC_ by gadolinium following excitotoxicity attenuated sustained calcium influx and prevented neuronal death. Additional studies, including *in vivo *experiments, are needed to clarify the characteristics, structure, and exact function of this channel in neurons following cerebral ischemia.

### 2.7. Intracellular Calcium Sequestering and Release: Release from Mitochondria and Endoplasmic Reticulum

#### 2.7.1. Mitochondria

In excitable cells such as neurons, mitochondria play a role in regulating intracellular calcium levels [75, 76]. In neurons, one way this is achieved is through the exchange of calcium ions from the matrix for cytosolic sodium ions via the mitochondrial sodium/calcium exchanger (NCX_MITO_), located in the inner mitochondrial membrane [77, 78]. There is also an interplay between NMDA-induced calcium stimulation, mitochondrial sequestering, and NCX_MITO_ suggesting that calcium is recycled across the mitochondrial membrane of neurons via the NCX_MITO_ in response to overstimulation of the NMDA receptors.

However, mitochondria have a limit to the amount of calcium that can be sequestered and this limitation is also influenced by the metabolic status of the cell. Thus, while mitochondrial calcium sequestration is a protective response, once this system becomes overwhelmed, it has severe consequences for the cell, leading to the activation of proapoptotic proteins and eventual cell death. Therefore following cerebral ischemia, the inability of mitochondria to adequately buffer neuronal intracellular calcium can result in calcium dysregulation [7, 79]. In addition, mitochondria can undergo other changes, which are damaging to the cell such as the loss of the mitochondrial membrane potential, increased mitochondrial membrane permeability, release of cytochrome C (a proapoptotic protein which in turn causes release of ER calcium stores) and excessive reactive oxygen species production [80].

#### 2.7.2. Endoplasmic Reticulum

The ER serves as a storage facility for calcium in neurons and other cells. The ER plays a fundamental homeostatic role during and following cerebral ischemia, by sequestering excess cytosolic calcium, which is thought to prevent ER stress and thus provide a protective mechanism against cell death [8]. However, when overwhelmed, ER homeostasis becomes dysregulated, resulting in subsequent calcium release, which contributes to calcium-associated cell death processes [81]. Normally, ER calcium influx is controlled by the Ca^2+^-ATPase pump located on the ER membrane, but during and following ischemia, its function in neurons is compromised due to declining ATP levels. Furthermore, cerebral ischemia activates phospholipase C causing hydrolysis of PIP2 (phosphatidylinositol (4, 5) bisphosphate) to release the signalling molecule IP3 (inositol (1-,4-,5-) trisphosphate). Receptors for IP3 (inositol (1-,4-,5-)trisphosphate receptors IP_3_R_s_) are located on the ER membrane and function as ligand-gated channels [81, 82]. Activation of P_3_R_s_ by IP3 results in a rapid release of calcium from the ER. Similarly, ryanodine receptor (R_y_R_s_) calcium release channels located on ER membranes are also likely to be activated following cerebral ischemia resulting in additional release of calcium via these channels [82].

### 2.8. Store-Operated Intracellular Calcium Entry

Store-operated calcium entry (also called capacitative calcium entry) refers to an influx of extracellular calcium across the plasma membrane via store-operated calcium channels (e.g., ORAI, TRP channels) in response to ER intracellular calcium release and store depletion [83]. Recently, this calcium entry mechanism has been demonstrated to occur following cerebral ischemia and contribute to neuronal death [84]. In neurons, the activation of store-operated channels is regulated by the ER transmembrane sensing protein STIM2 (stromal interaction molecule), which when activated interacts with and stimulates store-operated calcium channels. Berna-Erro et al. [84] have shown that neurons from STIM2 knockout mice are more resistant to hypoxia, and that the mice display less neurological damage following focal ischemia.

## 3. Neuronal Intracellular Calcium Homeostatic Mechanisms following Cerebral Ischemia

### 3.1. Calcium ATP-ase Pump

The calcium ATPase pump (or Plasma Membrane Calcium ATPase pump; PMCA) serves to regulate intracellular calcium levels by actively expelling calcium out of the cell. It has a high affinity but low transporting capacity for calcium. Its structure consists of ten transmembrane domains which forms the calcium-permeable pore and three intracellular loops which regulate its activity [85]. It is driven by ATP, with one calcium ion being removed for every ATP molecule hydrolysed. There are four PMCA isoforms (PMCA 1-4) with PMCA2 and PMCA3, mostly confined to the brain. During cerebral ischemia, reduced ATP generation results in compromised PMCA activity [86]. In addition, ischemia-induced caspase activation can cleave and inactive PMCA in neuronal cells allowing calcium overload [87]. Therefore, while the PMCA is an important neuronal calcium extrusion mechanism, its activity can be severely impeded by the intracellular biochemical changes that occur in neurons following ischemia.

### 3.2. Sodium Calcium Exchanger (Calcium Exit Mode)

Under normal physiological conditions NCX acts as a calcium extrusion transporter by operating in the forward or calcium exit mode [88]. As mentioned above, while there may be circumstances when it operates in calcium entry mode in neurons following cerebral ischemia; it is likely to be the major calcium efflux mechanism in cells that recover from the initial ischemia insult and in which the exchanger has not been severely inactivated by calpain cleavage [74]. For example, it has been shown that overexpression of the calpain resistant NCX2 isoform, but not calpain sensitive NCX3 isoform in cerebellar granule neuronal cultures reduces glutamate-induced calcium influx and neuronal death [89]. The beneficial effects of NCX activity following cerebral ischemia are further supported by data showing that NCX knockout mice suffer more brain injury following both global and focal cerebral ischemia [72, 73].

## 4. Summary

Excessive intracellular calcium influx is a major instigator of neuronal cell death following cerebral ischemia. This influx is mediated by a number of important channels and transporters. In addition, the overload of calcium in intracellular stores and the subsequent release of calcium from these stores can further exacerbate the problem. In contrast, there are only two known mechanisms to allow calcium exit (PMCA and NCX), both of which are susceptible to biochemical and/or proteolytic inactivation caused by intracellular disturbances associated with ischemia. This imbalance proves to be a major downfall in a neuron's ability to maintain calcium homeostasis following ischemia, which ultimately contributes to its demise. 

While researchers have made significant progress in understanding these important calcium influx and efflux pathways, especially under pathological conditions such as cerebral ischemia, it has not translated into any clinical therapeutic neuroprotective agents. However, it is anticipated that further investigation of calcium influx and efflux pathways will eventually enable the design of drugs to manipulate neuronal intracellular calcium levels following ischemia and lead to new neuroprotective therapies.
